# Intraoperative Hypotension Prediction—A Proactive Perioperative Hemodynamic Management—A Literature Review

**DOI:** 10.3390/medicina59030491

**Published:** 2023-03-02

**Authors:** Jakub Szrama, Agata Gradys, Tomasz Bartkowiak, Amadeusz Woźniak, Krzysztof Kusza, Zsolt Molnar

**Affiliations:** 1 Department of Anesthesiology, Intensive Therapy and Pain Management, Poznan University of Medical Sciences, 60-355 Poznan, Poland; 2 Department of Anesthesiology and Intensive Therapy, Semmelweis University, 1085 Budapest, Hungary

**Keywords:** hypotension, perioperative, prediction, hypotension prediction index, hemodynamic monitoring

## Abstract

Intraoperative hypotension (IH) is a frequent phenomenon affecting a substantial number of patients undergoing general anesthesia. The occurrence of IH is related to significant perioperative complications, including kidney failure, myocardial injury, and even increased mortality. Despite advanced hemodynamic monitoring and protocols utilizing goal directed therapy, our management is still reactive; we intervene when the episode of hypotension has already occurred. This literature review evaluated the Hypotension Prediction Index (HPI), which is designed to predict and reduce the incidence of IH. The HPI algorithm is based on a machine learning algorithm that analyzes the arterial pressure waveform as an input and the occurrence of hypotension with MAP <65 mmHg for at least 1 min as an output. There are several studies, both retrospective and prospective, showing a significant reduction in IH episodes with the use of the HPI algorithm. However, the level of evidence on the use of HPI remains very low, and further studies are needed to show the benefits of this algorithm on perioperative outcomes.

## 1. Introduction

Each year, around 230 million surgical procedures are performed worldwide, resulting in a significant number of patients suffering from possible perioperative complications [1]. Surgical procedures performed in high-risk patients constitute less than 15% of all of these procedures; however, such patients account for a substantial number of intensive care unit (ICU) admissions in the perioperative period, remain the main cause of prolonged recovery, and are responsible for more than 80% of perioperative deaths [1,2]. The term “high risk” is not clearly defined, and many factors can influence the rate of complications, including advanced age and comorbidities and the complexity and urgency of the procedure, [1,2]. 

The cornerstone of perioperative management is to maintain hemodynamic stability, including the avoidance or reduction of the rate of intraoperative hypotension (IH) [3]. Intraoperative hypotension is responsible for diminished organ perfusion and is associated with perioperative morbidity and mortality [3,4,5,6,7,8,9,10,11,12,13,14,15]. In order to avoid IH and cardiovascular instability, monitoring the patient’s hemodynamics appears to be an essential element of appropriate anesthetic management. However, recent meta-analyses on perioperative goal directed therapy and hemodynamic monitoring showed various effects on postoperative complications and did not show any benefit in reducing mortality [16,17,18].

The newly developed machine learning algorithm, the Hypotension Prediction Index (HPI), is designed to advise the clinician of an upcoming hypotensive event before it occurs. Furthermore, by implementing various hemodynamic monitoring parameters, it might provide information about the most probable cause of hypotension and aid in making treatment decisions. A narrative review on the HPI technology was published in 2022, which included validation studies, retrospective cohorts, studies performed in the intensive care unit (ICU) and prospective randomized trials [19]. The aim of this literature review is to focus on the most important clinical studies and the limitations and future directions of the HPI algorithm. 

## 2. Intraoperative Hypotension—Definition and Incidence

Despite substantial literature on the subject of adverse effects related to intraoperative hypotension, its definition is poorly defined. As mean arterial pressure (MAP) is an accurate measure of the arterial blood pressure during the whole cardiac cycle and is the driving force of organ perfusion, the most widely used definition of IH is MAP below 65 mmHg [4,20]. However, a recent literature review found 140 different definitions of IH in 130 articles [21]. Some of the definitions used absolute threshold values, as systolic blood pressure (SBP) <100 mmHg or below 80 mmHg. Other frequently used definitions include decline in SBP >20% below baseline and a combination of SBP <100 mmHg and/or 30% below baseline [20,21]. However, the issue of “baseline” blood pressure appears with these definitions, as there is a question about which blood pressure should be considered the baseline [3,4]. Typically, preinduction blood pressure or a single measurement in the preoperative ward were considered the baseline. A study evaluating the relationship between preinduction and preoperative blood pressure in more than 4400 patients showed higher preinduction values compared to the measurement outside the operating theatre with high variability among patients and within the patients [22]. Furthermore, there is a weak correlation between preinduction blood pressure measurements and the blood pressure measured in an ambulatory setting outside the hospital environment, so the perioperative values of blood pressure give a poor estimate of true baseline patient blood pressure [23]. Due to the wide range of intraoperative hypotension definitions, the rate of this event can vary from 5 to 99%, depending on the definition used [21]. Taking into account that most of the perioperative measurements are taken noninvasively, usually in 5 min intervals, the true incidence of intraoperative hypotension may be underestimated [4]. 

## 3. Adverse Events Related to Intraoperative Hypotension

The association between IH and end-organ injury has been documented in many studies. Even a short period of hypotension can be related to an increased risk of myocardial injury [7,8,24,25], acute kidney injury [7,8,9], postoperative stroke [15] and postoperative mortality [12,13]. Both the duration and the severity of IH can affect the rate of postoperative organ injury [7,8]. A large meta-analysis from 2018 including 14 studies on the adverse outcomes of IH revealed an association between IH and an increased risk of major cardiac events and 30-day postoperative mortality in a group of more than 120,000 patients [26]. A study performed in a group of 33,000 patients undergoing noncardiac surgery showed that the odds ratio (OR) for kidney and myocardial injury with even short 1–5 min episodes of severe hypotension defined as MAP <55 mmHg was 1.18 (1.06–1.31) and 1.30 (1.06–1.50), respectively [7].

## 4. Prevention of Hypotension by the Hypotension Prediction Index

Even with the use of hemodynamic monitoring devices and goal-directed perioperative therapy, the management of IH takes place when cardiovascular instability has already occurred. It was demonstrated in a study including 255 patients undergoing major surgery that neither MAP, heart pulse rate, stroke volume, or stroke volume variation are able to predict the occurrence of IH [27].

From the physiological point of view, hypotensive events start even before they can be observed by the attending physician. Measurement of the changes in compensatory reactions and variability of hemodynamic parameters in the early stages of cardiovascular instability might allow an early identification of IH, even before it occurs [3]. The hypotension prediction algorithm is based on the complex and detailed analysis of hemodynamic features extracted from the arterial pressure waveform with the use of machine learning methods able to construct mathematical models for the prediction of hemodynamic instability, or IH [3]. In machine learning, an algorithm is developed that uses multiple input variables to associate them with the output variable [4]. The commercially available hypotension prediction index (HPI; Edwards Lifesciences, Irvine, CA, USA) algorithm is based on such a machine learning algorithm with the complex analysis of the arterial pressure waveform as an input and the occurrence of hypotension with MAP <65 mmHg for at least 1 min as an output. The HPI algorithm gives the clinician an unitless number, ranging from 0–100, informing about the likelihood that within 5–15 min a hypotensive event will occur despite the patients being still hemodynamically stable [4]. The higher the value of the HPI, the greater the risk of hypotensive events. The algorithm was developed by analyzing the arterial pressure waveforms of 1334 mixed OR/ICU patients experiencing more than 25,000 hypotensive events [28]. Within each arterial pressure waveform, several subphases can be analyzed, which are related to some physiological parameters such as contractility, stroke volume, and aortic compliance. The analysis of the parameters derived from the arterial waveform allowed the machine learning devices to generate a hypotensive prediction algorithm [3]. The algorithm was validated both internally and externally in patients undergoing major surgery and in patients in the intensive care unit [27,28]. The sensitivity and specificity of predicting hypotension with this model are 92% and 92% at 5 min, 89% and 90% at 10 min, and 88% and 87% at 15 min, respectively, before the occurrence of hypotension [4,28]. However, there are studies reporting much lower sensitivity and specificity, with values of 62.4% and 77.7%, respectively [25,29].

**Figure 1:**
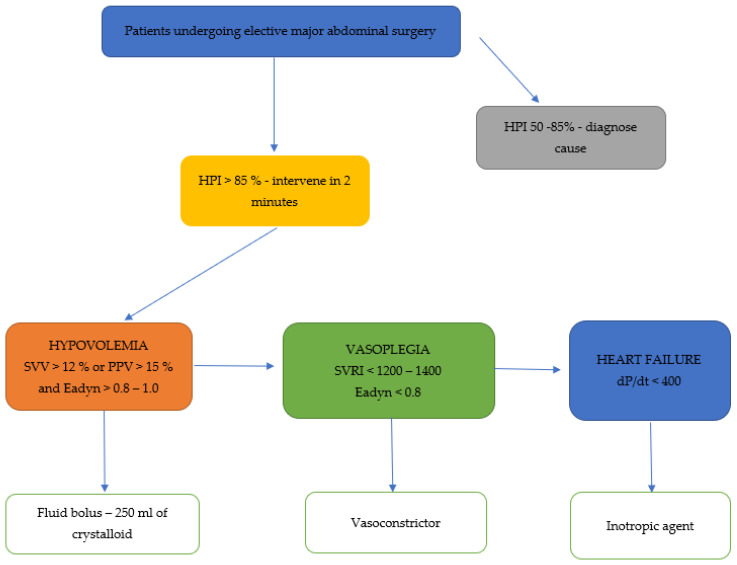
An example of a hemodynamic protocol based on HPI derived parameters.

Along with the HPI algorithm, the HemoSphere monitor has the option of a secondary screen that provides information on the preload, contractility, and afterload, as well as the possible cause of a future hypotensive event [4]. To determine preload, the algorithm includes SVV as well as a well-established dynamic preload variable assessing fluid responsiveness. In patients assessed as fluid responders with SVV values >12%, fluid administration will likely increase stroke volume and cardiac output [30,31]. Another variable helpful in optimizing the hemodynamics is dP/dt, a variable determining cardiac contractility. There is a correlation between dP/dt values obtained from the arterial pressure waveform and dP/dt measured invasively by left ventricular catheterization in animal studies and also in anesthetized patients undergoing coronary artery bypass surgery [32,33,34]. Finally, the third element is the dynamic arterial elastance (Eadyn), which is calculated by the quotient of pulse pressure variation and stroke volume variation (PPV/SSV). The optimal cut-off value for Eadyn is 1.0, with lower values suggesting a rather poor response to fluid administration and advising the use of vasopressors. On the contrary, with high Eadyn values, one would expect beneficial blood pressure increases with boluses of fluids. Figure 1 presents an example of a hemodynamic protocol including all the available HPI platform derived parameters.

## 5. HPI Clinical Studies

Along with the introduction of this technology, clinical trials started to appear evaluating the effectiveness of the algorithm in clinical practice [35,36,37,38,39,40,41]. The summary of the main studies, including the HPI algorithm, is presented in Table 1. One of the first trials was a prospective, randomized study evaluating the effect of HPI monitoring on the incidence of hypotension in patients undergoing total hip arthroplasty under general anesthesia. The HPI group had a significantly reduced incidence of IH, measured by the number of hypotensive events per hour and the reduced duration of IH in relation to total anesthesia time [35]. The HYPE randomized clinical trial published in 2020 analyzed the effect of the HPI algorithm on the incidence of IH in patients undergoing elective noncardiac surgery. The main outcome of the study was the time-weighted average (TWA) of hypotension during surgery, which is calculated as the depth of hypotension below a MAP of 65 mmHg × time spent below a MAP of 65 mmHg divided by the total duration of surgery. The study showed that in the HPI group, the time-weighted average of hypotension was 0.10 mmHg in comparison to 0.44 mmHg in the standard group, with a median difference of 0.38 mmHg (*p*-value = 0.001) [36]. 

A pilot randomized trial enrolled 214 patients having moderate- or high-risk noncardiac surgery into two groups: one group receiving HPI guided hemodynamic management and the other receiving conventional management. The authors found no significant difference in the TWA of MAP <65 mmHg in both studied populations. A more detailed analysis of the study revealed that almost half of the HPI alerts >85 indicating prompt intervention were ignored by the clinicians due to short warning times, complex treatment algorithms, or choosing the “observe” (no intervention) option. A post hoc analysis restricted to the situations where the clinicians made an intervention according to the HPI algorithm revealed a 57% reduction in the episodes of IH [37]. A retrospective analysis of 100 patients undergoing moderate or high-risk surgery with invasive blood pressure monitoring compared the incidence of IH in patients receiving either standard APCO monitoring (with the Flotrac) or HPI guided management. In the FloTrac group, 84% of patients experienced episodes of hypotension, while in the HPI group, only 52% of patients were hypotensive (*p*-value = 0.001). The TWA of hypotension below 65 mmHg was 0.27 mmHg in the FloTrac group and 0.10 mmHg in the HPI group (*p* = 0.001). The median duration of each hypotensive event with MAP <65 mmHg was 2.75 min in the FloTrac group and 1 min in the HPI group (*p* = 0.002) [40]. Another retrospective single-center study on 104 patients undergoing urgent or elective non-cardiac surgery with a moderate to high risk of bleeding compared the HPI technology with the use of APCO derived goal-directed therapy. The TWA of hypotension <65 mmHg was significantly (*p* = 0.037) lower in the HPI group in comparison to the APCO group: 0.09 mmHg vs. 0.23 mmHg, respectively. The patients in the HPI group had a median 2-day shorter stay in the hospital (*p* = 0.019) [41].

### Limitations of the HPI Technology

Although the majority of the studies show promising results on the effect of HPI on the reduction of IH, there are a few issues that still need some further analysis. A study published in 2019 conducted in patients undergoing major cardiac and vascular surgery showed the HPI technology predicted IH with a sensitivity and specificity of 62.4% and 77.7%, respectively, five to seven minutes before the occurrence [29]. The results of the study suggested that HPI had a high negative predictive value, which meant that low HPI values can establish a “safe zone,” where patients are at low risk of developing hypotension. On the other hand, the positive predictive value was very low, showing that high HPI values were not predictive enough to prompt an intervention to prevent hypotension [29]. 

As the HPI algorithm uses the APCO technology, all situations where the APCO parameters are less reliable will affect the use of the HPI technology [42,43]. The quality of the arterial pressure waveform may depend on the site of cannulation, and discrepancies between central and peripheral blood pressures have been described during deep hypothermic circulatory arrest, cardiopulmonary resuscitation, in patients with septic shock receiving high doses of vasoconstrictors, and in patients during reperfusion after liver transplant [44]. Femoral arterial pressures were more reliable than radial arterial pressures in patients after weaning from CPB [45]. The arterial waveform can be affected by cardiac pathologies, including aortic stenosis, aortic insufficiency, and atrial fibrillation, making the APCO derived values unreliable [43]. 

Further, the hemodynamic protocols that HPI is based on, including SVV and fluid responsiveness, are reported to be unreliable in both spontaneously breathing patients and during mechanical ventilation with low tidal volumes, irregular heartbeats, increased abdominal pressure, and an open thorax [45]. 

Furthermore, several studies have questioned the application of the pulse contour technology in patients undergoing major cardiac, vascular, and thoracic surgery [46,47,48,49]. The APCO technology has been shown to have low accuracy in patients with a low cardiac index due to alterations in systemic vascular resistance [46]. A study comparing four arterial pressure analysis techniques for cardiac output measurement in patients after elective coronary artery bypass surgery showed their poor accuracy, precision, and trending ability in comparison with cardiac output measured with the intermittent transpulmonary thermodilution technique [50]. Some have also noticed that the HPI alarm appeared on the monitor screen just two minutes before the occurrence of IH, which is an inadequate amount of time to make an intervention to prevent the occurrence of hypotension [4]. Most of the trials used the HPI technology along with some specific therapeutic algorithms, which might cause a bias whether the benefits on prevention hypotension are due to technology itself or due to application of the algorithm. The algorithm itself might also be a problematic issue, as demonstrated in the Maeheshari study [37], where the clinicians did not follow the algorithm, considering it to be too complicated. 

## 6. The HPI Future Directions

The majority of the presented literature shows that the HPI technology has the potential to reduce the rate and cumulative duration of intraoperative hypotension. However, there are few more questions that need to be answered before implementing the technology to an everyday basic hemodynamic monitoring. The technology still needs further research in different clinical scenarios, with a special interest in cardiac and major vascular surgery. Furthermore, it needs to be determined whether the application of the HPI algorithm may reduce the rate of perioperative mortality and morbidity and reduce the costs to the healthcare system secondary to these adverse outcomes. The future of HPI in the operating room might also be affected by human–computer interaction and the clinicians’ acceptance of the presence and decision making capabilities of the artificial intelligence devices. Finally, the HPI technology might become an element of the personalized perioperative hemodynamic management protocols; however, these topics also need further study.

## 7. Conclusions

Intraoperative hypotension remains a frequent phenomenon and is related to increased perioperative morbidity and mortality. The published studies show some promising results on the substantial reduction of the incidence and total duration of IH with the HPI algorithm. However, there are still many limitations to the technology itself and its application to various clinical situations. Furthermore, the value of the HPI algorithm as an element of everyday clinical practice and its role in decreasing perioperative organ injury remain to be established.

## Figures and Tables

**Table 1:** Summary of the main HPI studies.

Study, Year of Publication	Study Type	Number of Patients	Study Group	Primary Outcome	Main Results
Schneck et al., 2019 [35]	Randomised prospective interventional trial	Total 99 patients—25 HPI, 50 historic control, 25 standard care,	Primary hip arthroplasty under general anesthesia	Frequency (n/hour), absolute and relative hypotension (defined as MAP < 65 mmHg for more than 1 min) duration (% of total anesthesia time)	Significant reduction of intraoperative hypotension in the HPI group (percentage of patients with at least one episode of hypotension; 48% vs. 80% and 87%, in the HPI, historic control and control group, respectively). Significant reduction of number of hypotensive episodes (HPI 0 (0–1), historic control 2 (1–3), and control group 5 (2–6); median value (interquartile range), *p* < 0.001) and duration of hypotension—minutes below MAP 65 mmHg in % of total anesthesia time (HPI 0 (0–1), historic control 7 (2–17) and control group 6 (2–12); median value (interquartile range) *p* < 0.001).
Wijnberge et al.,2020 [36]	Prospective randomized controlled trial	Total 68 (HPI = 34, standard care = 34)	Elective non cardiac surgery	TWA of hypotension with MAP < 65 mmHg	The median TWA of hypotension was 0.10 mmHg in the HPI group vs. 0.44 mmHg in the standard therapy group (*p* = 0.001). The median duration of hypotension per patient was 8.0 min in the HPI group vs. 32.7 min in the standard therapy group (*p* < 0.001).
Maheshwari et al.,2020 [37]	Prospective randomized controlled trial	Total 214 (HPI 105, standard care = 109)	Moderate or high-risk non cardiac surgery	TWA of hypotension with MAP < 65 mmHg	The median TWA of hypotension was 0.14 mmHg in the HPI group vs. 0.14 mmHg in the standard therapy group (*p* = 0.757).
Tsoumpa et al., 2021 [38]	Prospective randomized controlled trial	Total 99 (HPI = 49, standard care = 50)	Moderate or high-risk non cardiac surgery	TWA of hypotension with MAP < 65 mmHg	The median TWA of hypotension was 0.16 mmHg in the HPI group vs. 0.50 mmHg in the standard therapy group (*p* = 0.0003).
Murabito et al.,2022 [39]	Prospective randomized controlled trial	Total 40 (HPI = 20, standard care = 20)	Major general surgery	TWA of hypotension with MAP < 65 mmHg	The median TWA of hypotension was 0.12 mmHg in the HPI group vs. 0.37 mmHg in the standard therapy group (*p* = 0.025).
Grundmann et al., 2021 [40]	Retrospective observational study	Total 100 (HPI = 50, Flotrac = 50)	Moderate or high-risk non cardiac surgery	TWA of hypotension with MAP < 65 mmHg	The median TWA of hypotension was 0.10 mmHg in the HPI group vs. 0.27 mmHg in the standard therapy group (*p* = 0.001). In the HPI group 26 patients (52%) vs. 42 patients (84%) experienced a hypotension (*p* = 0.001).
Solares et al., 2022 [41]	Retrospective study	Total 104 (HPI = 52, Flotrac = 52)	Urgent or elective non cardiac surgery with moderate to high risk of bleeding	TWA of hypotension with MAP < 65 mmHg	The median TWA of hypotension was 0.09 mmHg in the HPI group vs. 0.23 mmHg in the Flotrac group (*p* = 0.037). Postoperative complications were less frequent in the HPI group (*p* = 0.035). Hospital length of stay was 2 days shorter in the HPI group (0.019).

## Data Availability

Not applicable.
